# Supplementary figures and images for: ‘Candidatus Phytoplasma asteris’ subgroups display distinct disease progression dynamics during the carrot growing season
Source: PLoS One. 2021 Feb 4;16(2):e0239956. doi: 10.1371/journal.pone.0239956 (PMC7861454; doi:10.1371/journal.pone.0239956)

**S1 Fig.** Experimental plot design


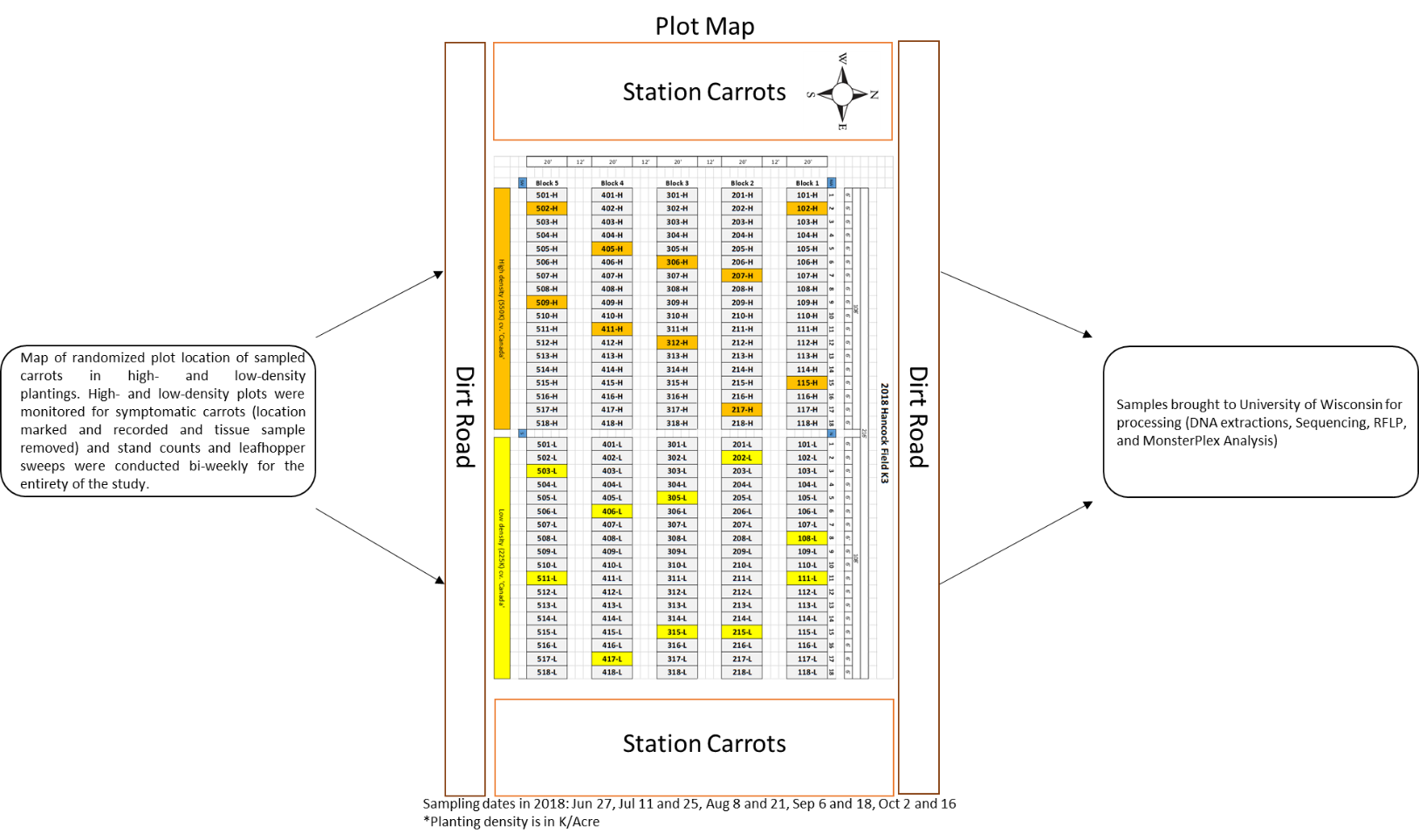

Supplement: S1 Fig — (DOCX) [file pone.0239956.s001.docx]
