# Supplementary material for: ‘Candidatus Phytoplasma asteris’ subgroups display distinct disease progression dynamics during the carrot growing season
Source: PLoS One. 2021 Feb 4;16(2):e0239956. doi: 10.1371/journal.pone.0239956 (PMC7861454; doi:10.1371/journal.pone.0239956)
Supplement: S2 Fig — (DOCX) [file pone.0239956.s002.docx]

**S2 Fig.** Diagnostic SNP’s used to differentiate subgroups 16SrI-A and 16SrI-B in the 16S rRNA gene sequence from A) Monsterplex sequencing and B) Nested PCR sequencing.

*SNP’s between subgroups used are highlighted in yellow

*Not all SNP’s between the subgroups were used

**A)**

16SrI-A 1 TGAGTAACGCGTAAGCAATCTACCCCTAAGACGAGGATAACAGTTGGAAACGACTGCTAA 60

16SrI-B 1 TGAGTAACGCGTAAGCAATCTGCCCCTAAGACGAGGATAACAGTTGGAAACGACTGCTAA 60

16SrI-A 61 GACTGGATAGTAGACAAGAAGGCATCTTCTTGTTTTTAAAAGACCTATTAATAGGTATGC 120

16SrI-B 61 GACTGGATAGGAGACAAGAGGGCATCTTCTTGTTTTTAAAAGACCTAGCAATAGGTATGC 120

16SrI-A 121 TTAGGGAGGAGCTTGCGTCACATTAGTTAGTTGGTGGGGTAAAGG 165

16SrI-B 121 TTAGGGAGGAGCTTGCGTCACATTAGTTAGTTGGTGGGGTAAAGG 165

**B)**

16SrI-A 1 GAAACGACTGCTAAGACTGGATAGTAGACAAGAAGGCATCTTCTTGTTTTTAAAAGACCT 60

16SrI-B 1 GAAACGACTGCTAAGACTGGATAGGAGACAAGAGGGCATCTTCTTGTTTTTAAAAGACCT 60

16SrI-A 61 ATTAATAGGTATGCTTAGGGAGGAGCTTGCGTCACATTAGTTAGTTGGTGGGGTAAAGGC 120

16SrI-B 61 AGCAATAGGTATGCTTAGGGAGGAGCTTGCGTCACATTAGTTAGTTGGTGGGGTAAAGGC 120

16SrI-A 121 CTACCAAGACTATGATGTGTAGCCGGGCTGAGAGGTTGAACGGCCACATTGGGACTGAGA 180

16SrI-B 121 CTAACAAGACTATGATGTGTAGCCGGGCTGAGAGGTTGAACGGCCACATTGGGACTGAGA 180

16SrI-A 181 CACGGCCCAAACTCCTACGGGAGGCAGCAGTAGGGAATTTTCGGCAATGGAGGAAACTCT 240

16SrI-B 181 CACGGCCCAAACTCCTACGGGAGGCAGCAGTAGGGAATTTTCGGCAATGGAGGAAACTCT 240

16SrI-A 241 GACCGAGCAACGCCGCGTGAACGATGAAGTATTTCGGTACGTAAAGTTCTTTTATTAGGG 300

16SrI-B 241 GACCGAGCAACGCCGCGTGAACGATGAAGTATTTCGGTACGTAAAGTTCTTTTATTAGGG 300

16SrI-A 301 AAGAATAAATGATGGAAAAATCATTCTGACGGTACCTAATGAATAAGCCCCGGCTAACTA 360

16SrI-B 301 AAGAATAAATGATGGAAAAATCATTCTGACGGTACCTAATGAATAAGCCCCGGCTAACTA 360

16SrI-A 361 TGTGCCAGCAGCCGCGGTAATACATAGGGGGCAAGCGTTATCCGGAATTATTGGGCGTAA 420

16SrI-B 361 TGTGCCAGCAGCCGCGGTAATACATAGGGGGCAAGCGTTATCCGGAATTATTGGGCGTAA 420

16SrI-A 421 AGGGTGCGTAGGCTGTTAAATAAGTTAATGGTCTAAGTGCAATGCTCAACATTGTGATGC 480

16SrI-B 421 AGGGTGCGTAGGCGGTTAAATAAGTTTATGGTCTAAGTGCAATGCTCAACATTGTGATGC 480

16SrI-A 481 TATAAAAACTGTTTAGCTAGAGTAAGATAGAGGCAAGTGGAATTCCATGTGTAGTGGTAA 540

16SrI-B 481 TATAAAAACTGTTTAGCTAGAGTAAGATAGAGGCAAGTGGAATTCCATGTGTAGTGGTAA 540

16SrI-A 541 AATGCGTAAATATATGGAGGAACACCAGTAGCGAAGGCGGCTTGCTGGGTCTTTACTGAC 600

16SrI-B 541 AATGCGTAAATATATGGAGGAACACCAGTAGCGAAGGCGGCTTGCTGGGTCTTTACTGAC 600

16SrI-A 601 GCTGAGGCACGAAAGCGTGGGGAGCAAACAGGATTAGATACCCTGGTAGTCCACGCCGTA 660

16SrI-B 601 GCTGAGGCACGAAAGCGTGGGGAGCAAACAGGATTAGATACCCTGGTAGTCCACGCCGTA 660

16SrI-A 661 AACGATGAGTACTAAACGTTGGGTAAAACCAGTGTTGAAGTTAACACATTAAGTACTCCG 720

16SrI-B 661 AACGATGAGTACTAAACGTTGGGTAAAACCAGTGTTGAAGTTAACACATTGAGTACTCCG 720

16SrI-A 721 CCTGAGTAGTACGTACGCAAGTATGAAACTTAAAGGAATTGACGGGACTCCGCACAAGCG 780

16SrI-B 721 CCTGAGTAGTACGTACGCAAGTATGAAACTTAAAGGAATTGACGGGACTCCGCACAAGCG 780

16SrI-A 781 GTGGATCATGTTGTTTAATTCGAAGGTACCCGAAAAACCTCACCAGGTCTTGACATGCTT 840

16SrI-B 781 GTGGATCATGTTGTTTAATTCGAAGGTACCCGAAAAACCTCACCAGGTCTTGACATGCTT 840

16SrI-A 841 CTGCAAAGCTGTAGAAACACAGTGGAGGTTATCAGTTGCACAGGTGGTGCATGGTTGTCG 900

16SrI-B 841 CTGCAAAGCTGTAGAAACACAGTGGAGGTTATCAGTTGCACAGGTGGTGCATGGTTGTCG 900

16SrI-A 901 TCAGCTCGTGTCGTGAGATGTTGGGTTAAGTCCCGCAACGAGCGCAACCCTTATTGTTAG 960

16SrI-B 901 TCAGCTCGTGTCGTGAGATGTTGGGTTAAGTCCCGCAACGAGCGCAACCCTTATTGTTAG 960

16SrI-A 961 TTGCCAGCACGTAATGGTGGGGACTTTAGCAAGACTGCCAGTGATAAATTGGAGGAAGGT 1020

16SrI-B 961 TTACCAGCACGTAATGGTAGGGACTTTAGCAAGACTGCCGGTGATAAATTGGAGGAAGGT 1020

16SrI-A 1021 GGGGACGACGTCAAATCATCATGCCCCTTATGACCTGGGCTACAAACGTGATACAATGGC 1080

16SrI-B 1021 GGGGACGACGTCAAATCATCATGCCCCTTATGACCTGGGCTACAAACGTGATACAATGGC 1080

16SrI-A 1081 TGTTACAAAGGGTAGCTGAAACGCAAGTTTTTGGCGAATCTCaaaaaaaCAGTCTCAGTT 1140

16SrI-B 1081 TGTTACAAAGGGTAGCTGAAGCGCAAGTTTTTGGCGAATCTCAAAAAAACAGTCTCAGTT 1140

16SrI-A 1141 CGGATTGAAGTCTGCAACTCGACTTCATGAAGTTGGAATCGCTAGTAATCGCGAATCAGC 1200

16SrI-B 1141 CGGATTGAAGTCTGCAACTCGACTTCATGAAGTTGGAATCGCTAGTAATCGCGAATCAGC 1200

16SrI-A 1201 ATGTCGCGGTGAATACGTTCTCGGGGTTTGTACACACCGCCCGTCA 1246

16SrI-B 1201 ATGTCGCGGTGAATACGTTCTCGGGGTTTGTACACACCGCCCGTCA 1246
