# Supplementary material for: ‘Candidatus Phytoplasma asteris’ subgroups display distinct disease progression dynamics during the carrot growing season
Source: PLoS One. 2021 Feb 4;16(2):e0239956. doi: 10.1371/journal.pone.0239956 (PMC7861454; doi:10.1371/journal.pone.0239956)
Supplement: S1 Table — (DOCX) [file pone.0239956.s003.docx]

**S1 Table.** A) Primers used to amplify 16S rRNA, B) MonsterPlex specific primers for amplification of effectors and 16S rRNA with amplicon size. Effector sequences were based on annotated reads from the Aster yellows witches'-broom phytoplasma complete genome CP000061.1. Primers were designed by aligning reads from the 16SrI-A genome and the 16SrI-B genome to determine SNPs that could be used to identify phytoplasma strain.

**A)**

|  | |  | |  |  |
| --- | --- | --- | --- | --- | --- |
|  | **Forward Primer (5’-3’)** | | **Reverse Primer (5’-3’)** | | |
| P1/P7 | AAGAGTTTGATCCTGGCTCAGG | | CGTCCTTCATCGGCTCTT | | |
| R16F2n/R16R2 | GAAACGACTGCTAAGACTGG | | TGACGGGCGGTGTGTACAAACCCCG | | |
|  |  | |  | | |

**B)**

|  | |  | |  | |  |  |  |
| --- | --- | --- | --- | --- | --- | --- | --- | --- |
|  | **Forward Primer (5’-3’)** | | **Reverse Primer (5’-3’)** | | **Amplicon (bp)** | | | |
| SAP05 | CCCTAATGAAGAGTTTGTTGGC | | TGCAAATTCTGCTATGTTTCCA | | 148 | | |  |
| SAP06 | GGTTGCAATTTCAAACACAATAGA | | TTCTTCTAGGACTGTTAGGTTTATTT | | 131 | | |  |
| SAP11 | CAAGTAATAGCTTCACCTAAAAAAG | | CAATTGAATATTCTTTAAATTCTTTATGTATTG | | 148 | | |  |
| SAP13 | CGACGCCTTATAATGTTCCTTTA | | TTTTTGCCATATCTTCTAATGATAAT | | 135 | | |  |
| SAP15 | GTGATGAAGAATTATACGCTGAT | | TGAATATTGTGTAATAAAGCTCCAATATA | | 139 | | |  |
| SAP19 | ACACTCCGTTGTATGCTTCTG | | ACGTCTTCTTCTATTGTTCGGATTA | | 149 | | |  |
| SAP21 | GCGATGGAAAAAGCAAATAATC | | GTTCTAATTTTATAATTTGTTGTACTTCATAG | | 172 | | |  |
| SAP27 | CGCACAATGCAACAAATATAC | | CAACATTATTAGGATTTATTGTTATCCC | | 165 | | |  |
| SAP35 | CGACTGCTCTGCCAACTATTA | | GAGCGTCTAATTTGGCATCAAC | | 159 | | |  |
| SAP36 | TGCAATCTGATTTAAATAGTGCAAC | | TTGAGGGTCTTGTAATTCTTGTTC | | 164 | | |  |
| SAP41 | GATTCAAGAGCATCAGAGGAACA | | CTCCGTTAGATGCAGATAACATAAATAA | | 103 | | |  |
| SAP42 | GTTATCCAGTTATGGCGATAAAT | | ATATCACCACTTAAATGATAAATATGAAT | | 136 | | |  |
| SAP44 | CATCAAGTAATGGCAATGGA | | TCGCCATATCTTCTAATGATAAA | | 145 | | |  |
| SAP45 | GAGTTATGGCAATGAATAATAATGAAG | | GTCGATCATATTGTAAAATTTCATCAG | | 144 | | |  |
| SAP48 | TGCATGCTTTCCCTGGTATAG | | AGTATCATTAAAAACGTTAGTTAATTC | | 101 | | |  |
| SAP49 | AATGATCTTTGTTGGCGTATCA | | CATTGCCCATTTCATTTAGATCTTC | | 124 | | |  |
| SAP54 | GGGATGGATAAAGATATTGCTAG | | GTTCATTATTTGATAATTGTTGTATTTCG | | 160 | | |  |
| SAP66 | AACAGTTATCCAACCAAATGAGTTTA | | TGGGTTGTTGCCCGATAAT | | 135 | | |  |
| SAP67 | ATGCCTTATATGTACAAGCAGAATTT | | TCATATGGCGTTGATAATTGATGATT | | 166 | | |  |
| SAP68 | GAAATGAATCGCGAACAAGCTATAA | | TTCGTGAGGCATAGCATTATGT | | 162 | | |  |
| 16S rRNA | TGAGTAACGCGTAAGCAATC | | CCTTTACCCCACCAACTAAC | | 165 | | |  |
|  |  | |  | |  | | |  |
