# Supplementary material for: ‘Candidatus Phytoplasma asteris’ subgroups display distinct disease progression dynamics during the carrot growing season
Source: PLoS One. 2021 Feb 4;16(2):e0239956. doi: 10.1371/journal.pone.0239956 (PMC7861454; doi:10.1371/journal.pone.0239956)
Supplement: S2 Table — (DOCX) [file pone.0239956.s004.docx]

### S2 Table. Effector abundance by subgroup. Effectors with a mean copy number of <1 were considered not present in that specific subgroup. Ratios of mean copy numbers are provided where the effector was present at copy numbers >1 for both subgroups.

|  | Mean copy number | | |  |  |  |  |
| --- | --- | --- | --- | --- | --- | --- | --- |
| Effector | 16SrI-A | 16SrI-B | Ratio | Assignment | Chi-sq | p-value | sig. |
| SAP05 | 13.9 | 6.0 | 2.3 | A&B | 82.7 | <.0001 | **** |
| SAP06 | 60.4 | 1.7 | 35.3 | A&B | 290.6 | <.0001 | **** |
| SAP11 | 16.4 | 0.3 |  | A-only | 305.4 | <.0001 | **** |
| SAP13 | 42.0 | 0.4 |  | A-only | 311.9 | <.0001 | **** |
| SAP15 | 59.6 | 0.4 |  | A-only | 316.1 | <.0001 | **** |
| SAP19 | 5.5 | 0.1 |  | A-only | 294.8 | <.0001 | **** |
| SAP21 | 0.0 | 0.0 |  | neither | 215.5 | <.0001 | **** |
| SAP27 | 1.2 | 1.1 | 1.1 | A&B | 1.4 | 0.2438 | NS |
| SAP35 | 2.5 | 1.9 | 1.3 | A&B | 10.7 | 0.0011 | ** |
| SAP36 | 0.6 | 0.6 |  | neither | 0.1 | 0.7411 | NS |
| SAP41 | 81.8 | 14.2 | 5.8 | A&B | 212.4 | <.0001 | **** |
| SAP42 | 78.3 | 17.4 | 4.5 | A&B | 181.3 | <.0001 | **** |
| SAP44 | 9.8 | 14.4 | 0.7 | A&B | 30.4 | <.0001 | **** |
| SAP45 | 23.0 | 0.5 |  | A-only | 300.9 | <.0001 | **** |
| SAP48 | 62.7 | 55.6 | 1.1 | A&B | 0.1 | 0.7929 | NS |
| SAP49 | 68.3 | 65.8 | 1.0 | A&B | 1.0 | 0.3198 | NS |
| SAP54 | 0.7 | 0.4 |  | neither | 25.7 | <.0001 | **** |
| SAP66 | 32.8 | 1.5 | 21.4 | A&B | 269.0 | <.0001 | **** |
| SAP67 | 0.5 | 0.0 |  | neither | 280.0 | <.0001 | **** |
| SAP68 | 2.2 | 0.1 |  | A-only | 290.5 | <.0001 | **** |
