# Supplementary material for: ‘Candidatus Phytoplasma asteris’ subgroups display distinct disease progression dynamics during the carrot growing season
Source: PLoS One. 2021 Feb 4;16(2):e0239956. doi: 10.1371/journal.pone.0239956 (PMC7861454; doi:10.1371/journal.pone.0239956)
Supplement: S3 Table — (DOCX) [file pone.0239956.s005.docx]

### S3 Table. Carrot stand counts for high and low planting density and AY incidence

| **Date** | **High-density plots** | | | | | | | | | **Low-density plots** | | | | | | | | |
| --- | --- | --- | --- | --- | --- | --- | --- | --- | --- | --- | --- | --- | --- | --- | --- | --- | --- | --- |
|  | **Stand ct.** | | | **%AY+ (edge)** | | | **%AY+ (interior)** | | | **Stand Ct.** | | | **%AY+ (edge)** | | | **%AY+ (interior)** | | |
|  | **Mean** | **±** | **SD** | **Mean** | **±** | **SD** | **Mean** | **±** | **SD** | **Mean** | **±** | **SD** | **Mean** | **±** | **SD** | **Mean** | **±** | **SD** |
| Jun 27 | 482 | ± | 78 | 0.0% | ± | 0.0% | 0.0% | ± | 0.0% | 175 | ± | 29 | 0.0% | ± | 0.0% | 0.0% | ± | 0.0% |
| Jul 11 | 495 | ± | 84 | 0.1% | ± | 0.1% | 0.0% | ± | 0.0% | 202 | ± | 40 | 0.0% | ± | 0.0% | 0.0% | ± | 0.0% |
| Jul 25 | 489 | ± | 78 | 0.2% | ± | 0.2% | 0.0% | ± | 0.0% | 204 | ± | 43 | 0.0% | ± | 0.0% | 0.1% | ± | 0.2% |
| Aug 8 | 487 | ± | 81 | 0.3% | ± | 0.3% | 0.0% | ± | 0.1% | 200 | ± | 38 | 0.7% | ± | 0.4% | 0.3% | ± | 0.3% |
| Aug 21 | 473 | ± | 64 | 0.4% | ± | 0.4% | 0.1% | ± | 0.2% | 204 | ± | 42 | 1.2% | ± | 0.8% | 0.9% | ± | 1.0% |
| Sep 6 | 491 | ± | 73 | 0.4% | ± | 0.4% | 0.1% | ± | 0.2% | 204 | ± | 40 | 1.5% | ± | 1.3% | 0.9% | ± | 0.9% |
| Sep 18 | 469 | ± | 78 | 0.8% | ± | 0.6% | 0.3% | ± | 0.3% | 195 | ± | 36 | 2.1% | ± | 1.8% | 1.6% | ± | 1.1% |
| Oct 2 | 471 | ± | 83 | 3.0% | ± | 1.5% | 2.1% | ± | 1.1% | 191 | ± | 38 | 8.1% | ± | 6.2% | 5.3% | ± | 1.4% |
| Oct 16 | 445 | ± | 83 | 4.5% | ± | 1.8% | 4.3% | ± | 1.9% | 185 | ± | 37 | 11.0% | ± | 5.9% | 8.2% | ± | 2.2% |
