# Supplementary material for: ‘Candidatus Phytoplasma asteris’ subgroups display distinct disease progression dynamics during the carrot growing season
Source: PLoS One. 2021 Feb 4;16(2):e0239956. doi: 10.1371/journal.pone.0239956 (PMC7861454; doi:10.1371/journal.pone.0239956)
Supplement: S4 Table — (DOCX) [file pone.0239956.s006.docx]

### S4 Table. Disease progression mixed model results

| Response | Effect Type | Source | df | F | P-value | Sig. |
| --- | --- | --- | --- | --- | --- | --- |
| fraction AY+ | fixed | *density* | 16 | 9.06 | 0.0083 | ** |
|  |  | *plot loc* | 16 | 2.63 | 0.1244 | NS |
|  |  | *week* | 157 | 479.49 | <.0001 | **** |
|  |  | *density*plot loc* | 16 | 0.01 | 0.9129 | NS |
|  |  | *density*week* | 157 | 26.81 | <.0001 | **** |
|  |  | *plot loc*week* | 157 | 1.97 | 0.1628 | NS |
|  |  |  |  |  |  |  |
|  | random | *plot* |  |  | 0.0395 | * |
|  |  |  |  |  |  |  |
| number AY+ | fixed | *density* | 16 | 0.17 | 0.69 | NS |
|  |  | *plot loc* | 16 | 3.06 | 0.1 | NS |
|  |  | *week* | 157 | 497.82 | <.0001 | **** |
|  |  | *density*plot loc* | 16 | 0.46 | 0.51 | NS |
|  |  | *density*week* | 157 | 0.07 | 0.79 | NS |
|  |  | *plot loc*week* | 157 | 1.02 | 0.31 | NS |
|  |  |  |  |  |  |  |
|  | random | *plot* |  |  | 0.05 | NS |
